# A study of clinical and molecular characteristics in bilateral primary breast cancer

**DOI:** 10.1002/cam4.6226

**Published:** 2023-06-09

**Authors:** Bin Li, Weiqi Xu, Jianing Cao, Duancheng Guo, Zhonghua Tao, Juan Jin, Xichun Hu

**Affiliations:** ^1^ Department of Breast and Urologic Medical Oncology Fudan University Shanghai Cancer Center Shanghai China; ^2^ Department of Oncology, Shanghai Medical College Fudan University Shanghai China; ^3^ Department of Hepatic Surgery Fudan University Shanghai Cancer Center Shanghai China

**Keywords:** bilateral primary breast cancer, clinicopathologic characteristics, mutation landscape, next‐generation sequencing

## Abstract

**Background:**

Bilateral primary breast cancer (BPBC) is a rare type of breast cancer. Studies on the clinicopathologic and molecular characteristics of BPBC in a metastatic context are very limited.

**Methods:**

A total of 574 unselected metastatic breast cancer patients with clinical information were enrolled in our next‐generation sequencing (NGS) database. Patients with BPBC from our NGS database were regarded as the study cohort. In addition, 1467 patients with BPBC and 2874 patients with unilateral breast cancer (UBC) from the Surveillance, Epidemiology, and End Results (SEER) public database were also analyzed to determine the characteristics of BPBC.

**Results:**

Among the 574 patients enrolled in our NGS database, 20 (3.5%) patients had bilateral disease, comprising 15 (75%) patients with synchronous bilateral disease and 5 (25%) patients with metachronous bilateral disease. Eight patients had bilateral hormone receptor‐positive (HR+)/human epidermal growth factor receptor‐negative (HER2−) tumors, and three had unilateral HR+/HER2− tumors. More HR+/HER2− tumors and lobular components were found in BPBC patients than in UBC patients. The molecular subtype of the metastatic lesions in three patients was inconsistent with either side of the primary lesions, which suggested the importance of rebiopsy. Strong correlations in clinicopathologic features between the left and right tumors in BPBC were exhibited in the SEER database. In our NGS database, only one BPBC patient was found with a pathogenic germline mutation in *BRCA2*. The top mutated somatic genes in BPBC patients were similar to those in UBC patients, including *TP53* (58.8% in BPBC and 60.6% in UBC) and *PI3KCA* (47.1% in BPBC and 35.9% in UBC).

**Conclusions:**

Our study suggested that BPBC may tend to be lobular carcinoma and have the HR+/HER2− subtype. Although our study did not find specific germline and somatic mutations in BPBC, more research is needed for verification.

## 1 INTRODUCTION

Breast cancer is the most common cancer among women, with a rising incidence in past decades.^1^ Bilateral primary breast cancer (BPBC) is a rare type of breast cancer that refers to the synchronous or metachronous occurrence of independent tumor lesions in the bilateral mammary glands and is classified as synchronous bilateral primary breast cancer (SBPBC) or metachronous bilateral primary breast cancer (MBPBC). Although the proportion of BPBC is quite small, according to the SEER database, it increased from 2.6% in 1975 to 7.5% in 2014.^2^ The studies on BPBC have not reported clear results, and no evidence‐based guidelines have been made for BPBC patients.^3^ Moreover, studies on the incidence and pattern of BPBC in Chinese women are more limited, particularly in patients developing metastatic disease.

At the genomic level, it was reported that *BRCA1/2* mutations were the most common germline mutations in 57 BPBC patients.^4^ In addition, among breast cancer patients with pathogenic mutations in *CHEK2*, approximately 20% of patients ultimately developed BPBC.^5^ However, current molecular studies of BPBC are very limited. In this study, we conducted a descriptive analysis of the phenotype, prognosis and molecular characteristics of metastatic BPBC in our next‐generation sequencing (NGS) database.

## 2 METHOD

### 2.1 Patients and cohorts

#### 2.1.1 FUSCC cohort

Our NGS database was built from the China‐Breast‐Umbrella study, in which 574 metastatic breast cancer patients underwent NGS of circulating tumor DNA (ctDNA) and biopsy in Fudan University Shanghai Cancer Center (FUSCC) from November 2017 to September 2021 and were treated with relevant targeted therapy based on the NGS results. Among these patients, 20 patients were extracted to characterize BPBC. The Ethics Committee of FUSCC has granted approval for this study (Approval number: 1705172–9). All patients provided written informed consent for the study.

The clinicopathologic information was obtained from our electronic medical record, including age of diagnosis, menopausal status, family history, pathologic result, Ki67 status, details of metastasis and previous treatments. The status of estrogen receptor (ER), progesterone receptor (PR), and human epidermal growth factor receptor 2 (HER2); Ki67 score; and other pathologic information were verified by the Department of Pathology of FUSCC. ER and PR positivity was defined as greater than or equal to 1% of tumor cells with positive nuclear staining, and HER2‐positive status was defined as 3+ in immunohistochemical (IHC) detection or 2+ in IHC combined with amplification of the HER2 gene by fluorescence in situ hybridization, according to the guidelines of the American Society of Clinical Oncology /College of American Pathologists.^6^, ^7^ Positive ER or PR referred to positive hormone receptor (HR). The proportion of ER‐positive and HER2‐positive status in patients with BPBC was calculated as the number of positive tumors in the total number of tumors.

#### 2.1.2 SEER database

We used the Surveillance, Epidemiology, and End Results (SEER) database to further compare the clinicopathologic differences between BPBC and unilateral breast cancer (UBC). Clinicopathologic data, including age of diagnosis, pathologic results, molecular subtype and survival information were obtained by SEER*Stat statistical software (https://seer.cancer.gov). We selected patients diagnosed with breast cancer from 2005 to 2015 with no distant metastasis. Patient ID (Identity Document) was used to merge the information, through which we excluded patients with multiple primary cancers and UBC. Finally, 1467 female patients with BPBC were included in our study. In addition, 2874 female UBC patients with no distant metastasis were randomly matched during the same diagnostic time from 2005 to 2015 via the R statistics package (R version 3.5.3; R: The R‐Project for Statistical Computing).

### 2.2 NGS data from the FUSCC cohort

ctDNA was extracted from the plasma of blood specimens and then sequenced using a QIAamp Circulating Nucleic Acid kit (Qiagen) for commercially available cancer‐associated gene panels in the laboratory of Burning Rock Biotech, as previously described.^8^ Tissue DNA was isolated from tissue samples by a QIAamp DNA FFPE tissue kit, as described in a previous study.^9^ Among 20 patients with BPBC, 10, 6 and 1 patients had NGS results from ctDNA for a panel of 520 cancer‐related genes, a panel of 108 cancer‐related genes and a panel of 168 cancer‐related genes, respectively; 12 patients had NGS results from metastatic tissues for the 520‐gene panel. In total, 11 patients had NGS results from both ctDNA and tissues. Among 554 patients with UBC, 419, 130 and 5 patients had NGS results from ctDNA for the 520‐gene panel, 108‐gene panel and 168‐gene panel, respectively. In addition, 337, 1 and 1 patients had NGS results from metastatic tissues for the 520‐gene panel, 108‐gene panel and 168‐gene panel, respectively. In total, 339 patients had NGS results from both ctDNA and tissues. Germline mutations were identified from white blood cells, and pathogenic/likely pathogenic variants were defined according to the American College of Medical Genetics and Genomics. In our study, only pathogenic/likely germline mutations were included in the analysis. Copy number variation (CNV) was analyzed with an algorithm based on the depth of sequencing data, and tumor mutation burden (TMB) was calculated per patient as the ratio between nonsynonymous variants and the size of the total coding region of the gene panel, according to previous studies.^8^, ^10^ The size of the total coding region for estimating TMB was 1.003 Mb in the 520‐gene panel, while TMB was not reported in the 108‐gene and 168‐gene panels.

To avoid bias, 62 common genes shared by all these panels were analyzed in this study. All genes included in these panels are recorded in Table S1, and the shared genes are highlighted.

### 2.3 Statistical analysis

The chi‐square test or Fisher's exact test was used to compare the categorical variables of clinicopathologic characteristics, while the unpaired *t* test was used to compare continuous variables. A nonparametric test (Mann–Whitney test) was used to compare TMB among different groups. Disease‐free survival (DFS) was defined as the time from surgery to local recurrence or distant metastasis. Overall survival (OS) was defined as the time from local recurrence or distant metastasis to death or the end of the follow‐up period. Survival was analyzed by the Kaplan–Meier (KM) method and log‐rank test.

Statistics were analyzed using GraphPad Prism 9.0. All data were analyzed by the R statistics package (R version 3.5.3; R: The R‐Project for Statistical Computing). All statistical tests were two‐sided, and the results were considered significant when the *p* < 0.05.

## 3 RESULTS

### 3.1 Disease presentation and characteristics of BPBC in our NGS database

A total of 574 patients of metastatic breast cancer with NGS data were eligible for analysis in our study, and among these patients, 20 (3.5%) patients were diagnosed with BPBC (Figure S1). We defined SBPBC as developing bilateral breast cancer within 6 months, and MBPBC was defined as the diagnosis of contralateral breast cancer more than 6 months after the first breast cancer diagnosis. In our database, 15 (75%) patients had SBPBC, and 5 (25%) patients had MBPBC. The median age at the first diagnosis in patients with BPBC was 49 years, with a range of 30–62 years. Among those 20 patients, 4 patients (20%) had a family history of tumors in first‐degree relatives. The clinicopathologic characteristics of BPBC patients in our NGS database are summarized in Table 1, and those of UBC patients are shown in Table S2. The most common type of tumor histology was invasive ductal carcinoma (IDC), regardless of the tumor side (left or right). Excluding 1 patient without pathology results, 12 patients had bilateral IDC, 2 patients had unilateral IDC and contralateral invasive lobular carcinoma (ILC), and 5 had unilateral IDC and contralateral ductal/lobular carcinoma in situ (DCIS/LCIS).

**TABLE 1:** Clinicopathologic characteristics of BPBC patients from FUSCC.

Characteristics	BPBC (*n* = 20)
Age (median, range)	49 (30–62)
Family history of cancers	
Yes	4 (20%)
No	11 (55%)
NA	5 (25%)
Breast surgery	
Yes	17 (85%)
No	3 (15%)
Histopathology	
No lobular component	15 (75%)
Lobular component	3 (15%)
NA	2 (10%)
The initial metastasis sites	
Liver	5 (25%)
Lung	7 (30%)
Bone	10 (50%)
The number of initial metastasis sites	
<3	17 (85%)
≥3	3 (15%)
ER positivity	62.5%
HER2 positivity	15%
Chemotherapy	
Adjuvant therapy	17 (85%)
Neoadjuvant therapy	1 (5%)

Abbreviation: NA: unknown or missing.

The correlations of clinicopathologic characteristics between left and right tumors were analyzed (Table 2), and the results suggested that molecular subtypes showed significant correlations between the left and right lesions (*p* < 0.05). In addition, eight patients had bilateral HR‐positive (HR+)/HER2‐negative (HER2−) tumors, and three patients had unilateral HR+/HER2− tumors. Two patients had bilateral triple‐negative tumors, five patients had unilateral triple‐negative tumors, and only six patients had unilateral HER2‐positive (HER2+) tumors (Table 3). The correlations of other clinicopathologic features had no statistical significance.

**TABLE 2:** Clinicopathologic features of left and right tumors in BPBC patients from FUSCC.

Clinicopathologic features	Left *n* = 20 (%)	Right *n* = 20 (%)	*p* (left vs. right)
Tumor histology			0.6747
DCIS	3 (15)	1 (5)	
LCIS	0 (0)	1 (5)	
IDC	15 (75)	15 (75)	
ILC	1 (5)	1 (5)	
NA	1 (5)	2 (10)	
Malignancy grade			0.2136
I	1 (5)	0 (0)	
II	5 (25)	6 (30)	
III	1 (5)	5 (25)	
NA	13 (65)	9 (45)	
TNM stage			0.3764
0/I	8 (40)	4 (20)	
II	3 (15)	8 (40)	
III	2 (10)	1 (5)	
IV	3 (15)	4 (20)	
NA	4 (20)	3 (15)	
Molecular subtype			**0.0342**
HR+/HER2−	10 (50)	10 (50)	
HR+/HER2+	0 (0)	2 (10)	
HR−/HER2+	0 (0)	4 (20)	
TNBC	6 (30)	4 (20)	
NA^a^	4 (20)	0 (0)	
Ki67 index			0.1399
< 20%	7 (35)	5 (25)	
≥ 20%	6 (30)	12 (60)	
NA	7 (35)	3 (15)	
Tumor size			0.5569
0–20 mm	8 (40)	7 (35)	
21–50 mm	4 (20)	7 (35)	
NA	8 (40)	6 (30)	
Nodal involvement			0.9307
Without	9 (45)	10 (50)	
With	6 (30)	5 (25)	
NA	5 (25)	5 (25)	

Abbreviations: DCIS, ductal carcinoma in situ; LCIS, lobular carcinoma in situ; IDC, invasive ductal carcinoma; ILC, invasive lobular carcinoma; NA, unknown; HR+, HR‐positive; HER2−, HER2‐negative; HER2+, HER2‐positive; HR−, HR‐negative; TNBC, triple‐negative breast cancer.

^a^ HR‐positive/HER2−unknown or HR/HER2 unknown.

**TABLE 3:** Pathologic phenotypes and mutation information of 20 BPBC patients from FUSCC.

Patient #	Laterality	Phenotype	Metastatic lesions	Mutant gene in ctDNA
1^a^	Left	HR+/HER2−	TNBC	CDK, PIK3R1, PTEN
	Right	HR+/HER2−		
2	Left	HR+/HER2−	Without data	ARID1A, FGFR1, TP53
	Right	HR+/HER2−		
3	Left	HR+/HER2 NA	HR−/HER2+	CCND2, ERBB2, MSH6, MYC, NF1, PIK3CA, TP53
	Right	HR+/HER2+		
4	Left	HR+/HER2−	HR+/HER2−	Without data
	Right	HR+/HER2−		
5	Left	HR+/HER2−	HR−/HER2+	TP53
	Right	HR−/HER2+		
6	Left	HR+/HER NA	HR+/HER2−	FGFR1, RB1, TP53
	Right	HR+/HER2−		
7	Left	HR+/HER2 NA	TNBC	KIT, PIK3CA, RAD51, TP53
	Right	TNBC		
8	Left	HR+/HER2−	Without data	APC, ATM, CBL, CHEK1, CHEK2, FBXW7, FGFR3, MET, MYC, NF1, PIK3R1, TP53
	Right	HR+/HER2−		
9	Left	TNBC	Without data	Negative
	Right	HR−/HER2+		
10	Left	HR+/HER2−	TNBC	Negative
	Right	HR+/HER2−		
11	Left	HR+/HER2−	HR−/HER2+	PIK3CA, TP53
	Right	HR−/HER2+		
12	Left	TNBC	Without data	AKT1, PIK3CA, ROS1, TP53
	Right	TNBC		
13	Left	HR+/HER2−	HR+/HER2−	NTRK1, PIK3CA
	Right	HR+/HER2−		
14	Left	TNBC	Without data	Without data
	Right	TNBC		
15	Left	TNBC	HR−/HER2+	APC, ARID1A, ERBB2, PIK3CA, SETD2, TP53
	Right	HR−/HER2+		
16	Left	HR+/HER2−	Without data	Negative
	Right	HR+/HER2−		
17	Left	TNBC	Without data	PIK3CA, TP53
	Right	HR+/HER2+		
18	Left	NA	TNBC	BRCA2
	Right	TNBC		
19	Left	HR+/HER2−	Without data	Without data
	Right	HR+/HER2−		
20	Left	TNBC	Without data	PIK3CA, SOX2, TP53
	Right	HR+/HER2−		

Abbreviations: ctDNA, circulating tumor DNA; HER2−, HER2‐negative; HER2+, HER2‐positive; HR−, HR‐negative; HR+, HR‐positive; NA, unknown; TNBC, triple‐negative breast cancer.

^a^ With germline *BRCA2* mutation.

Among the initial metastasis sites, bone, lung and liver were observed in 50%, 30% and 20% of BPBC patients, respectively. Eleven patients had pathologic data on metastatic lesions; among those, the molecular subtypes of eight patients were consistent with either side of the primary breast cancer. The molecular subtype of metastatic lesions in three patients was inconsistent with either side of the primary lesions, in which two patients presented with TNBC, although the primary lesions on both sides were HR+/HER2−, and one patient presented with an HR‐negative (HR−)/HER2+ tumor, but the primary lesions were HR+/HER2+ and HR+/HER2 unknown. Among patients with different molecular subtypes of primary breast cancers on the left and right sides, four patients also had pathologic data on metastatic lesions. Among those four patients, the molecular subtype in the metastatic lesions of three patient was HR−/HER2+, and that of the other patient was TNBC, consistent with the primary leisions on one side, which indicated that the metastatic lesions were associated with molecular subtypes with a more aggressive phenotype. It was also observed that among those four patients, the molecular phenotype of the metastatic lesions in three patients was related to the phenotype of the tumor with a higher clinical stage.

### 3.2 Mutation landscape and CNV profiles of patients with BPBC in FUSCC

**FIGURE 1:**
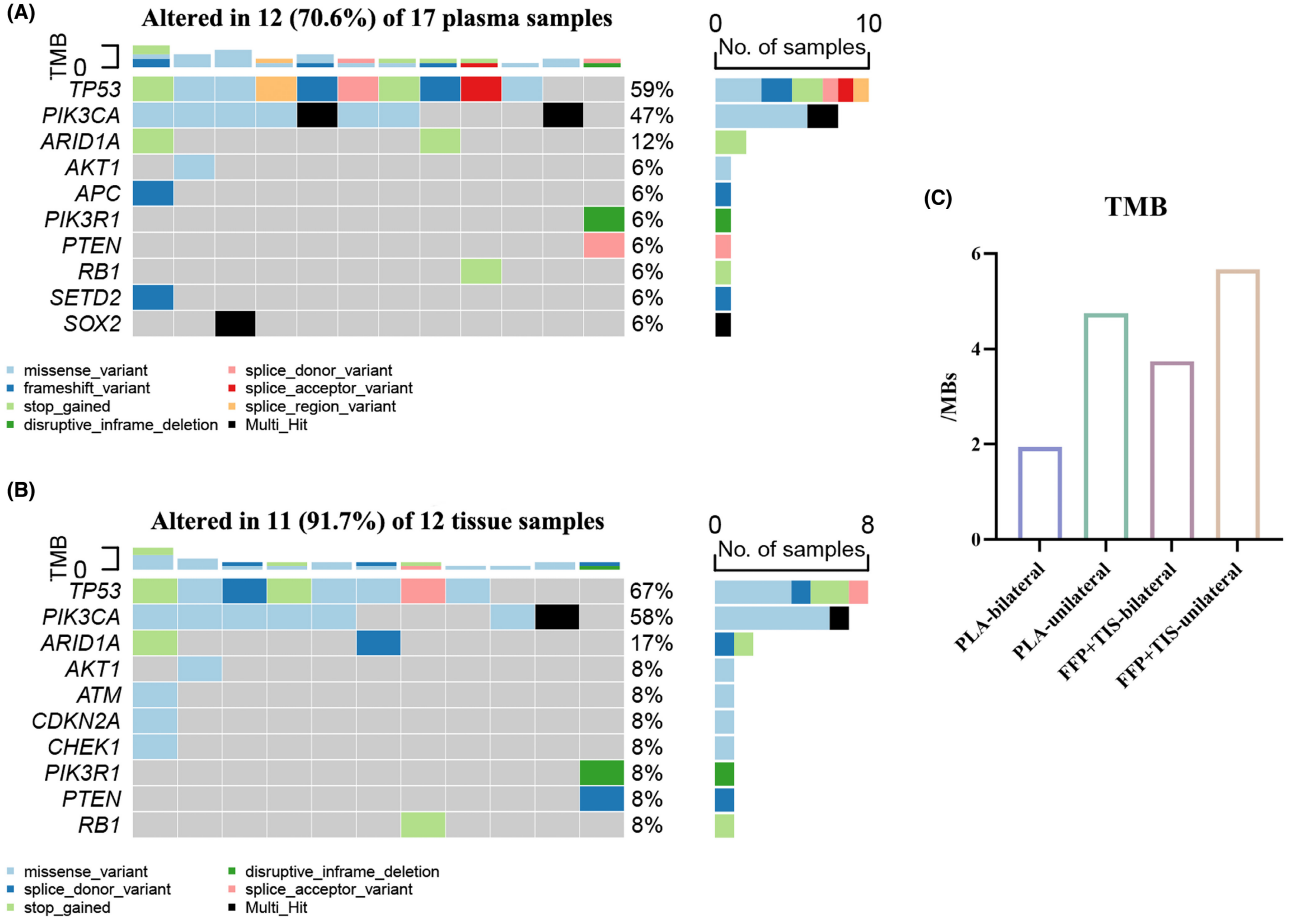
Mutations profiles of bilateral primary breast cancer (BPBC) patients in Fudan University Shanghai Cancer Center. (A) The distribution of the somatic mutations in plasma samples of BPBC patients. (B) The distribution of the somatic mutations in tissue samples of BPBC patients. (C) TMB among different subgroups in our next‐generation sequencing database. PLA, plasma samples; FFP + TIS, tissue samples.

Among 20 patients with BPBC, only one patient was found with a germline mutation in *BRCA2*. As shown in Figure S1, 17 patients with BPBC had NGS results from ctDNA, and 12 patients had NGS results from tissues of metastatic lesions. The distribution of the somatic mutations in available BPBC patients is shown in Figure 1A,B, and the somatic mutations in UBC patients are shown in Figure S2. The rates of *TP53* mutations were 58.8% and 66.7% in ctDNA and tissue in BPBC, respectively, and it was the most common mutation. *PIK3CA* and *ARID1A* mutations were also very common in BPBC, which was similar to UBC patients (Figure S2). We also analyzed TMB in different subgroups of our patients (Figure 1C).

For CNV profiles, BPBC patients were found to have the most copy number gains of 17q12 (*ERBB2*) in both plasma samples (11.8%, 2/17) and metastatic tissue samples (33.3%, 4/12), and the molecular type of the primary tumors on one side in those patients was HER2+. It was also observed that two patients had copy number gains of 8q24.21 (*MYC*) in plasma samples, and three patients had this amplification in metastatic tissue samples, of which one patient was found with TNBC on one side of primary tumor. Copy number gains of 17q11.2 (*NF1*) were found in two patients with plasma samples and two patients with metastatic tissue samples.

### 3.3 The clinicopathologic characteristics of patients with BPBC in SEER

**FIGURE 2:**
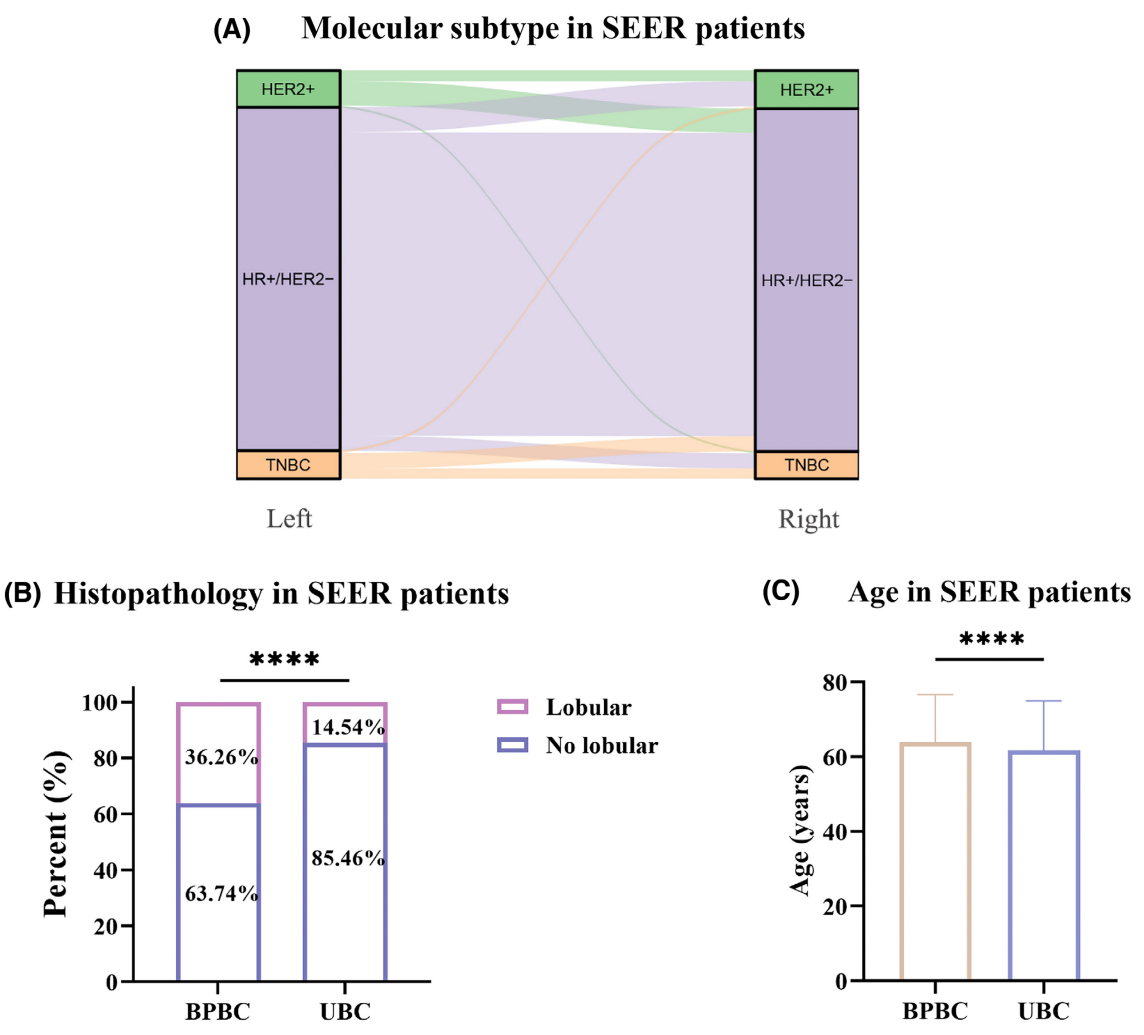
Clinicopathologic characteristics of bilateral primary breast cancer (BPBC) patients and unilateral breast cancer (UBC) patients from SEER. (A) Molecular subtype of left and right tumors of BPBC patients in SEER. (B) Histopathology of BPBC patients and UBC patients in SEER. (C) Age of BPBC patients and UBC patients in SEER. Lobular, with lobular component; No lobular, without lobular component; NA, unknown. * 0.01 ≤ p‐value <0.05, ** 0.001 ≤ p‐value <0.01, **** p‐value <0.0001.

Considering there were only 20 patients with BPBC in our NGS cohort, to characterize the clinicopathologic characteristics of patients with BPBC in depth, we collected data of BPBC patients from the SEER database for further analysis. A total of 1467 BPBC patients without metastasis from 2005 to 2015 were involved in the study, with a median age of 64 years. There were no differences in clinicopathologic characteristics between the left and right lesions, as shown in Table 4. Further analysis showed that clinicopathologic characteristics in the left tumors were significantly correlated with those in the right tumors (Table 5). As shown in Figure 2A, the molecular subtype of the left tumor had significant correlations with that of the right tumor. In the SEER database, 78.87% (1157/1467) of BPBC patients had the same molecular subtype in bilateral tumors.

**TABLE 4:** Clinicopathologic features of left and right tumors in BPBC from SEER.

Clinicopathologic features	Left *n* = 1467 (%)	Right *n* = 1467 (%)	*p* (left vs. right)
Tumor histology^a^			0.2930
IDC	961 (65.51)	958 (65.30)	
ILC	228 (15.54)	199 (13.57)	
IDC + ILC	122 (8.32)	135 (9.20)	
Malignancy grade^b^			0.1281
I	462 (31.49)	493 (33.61)	
II	675 (46.01)	654 (44.58)	
III	266 (18.13)	277 (18.88)	
TNM stage			0.1047
0/I	822 (56.03)	873 (59.51)	
II	477 (32.52)	453 (30.88)	
III	168 (11.45)	141 (9.61)	
Tumor size			0.2247
T0/Tis/T1	955 (65.10)	1001 (68.23)	
T2	387 (26.38)	363 (24.74)	
T3	85 (5.79)	74 (5.04)	
T4	40 (2.73)	29 (1.98)	
Nodal involvement			0.3569
N0	1084 (73.89)	1120 (76.35)	
N1	271 (18.47)	256 (17.45)	
N2	66 (4.50)	55 (3.75)	
N3	46 (3.14)	36 (2.45)	
Tumor location^c^			0.1137
Nipple	10 (0.68)	3 (0.20)	
Central portion	76 (5.18)	69 (4.70)	
Upper‐inner	213 (14.52)	164 (11.18)	
Lower‐inner	68 (4.64)	75 (5.11)	
Upper‐outer	442 (30.13)	458 (31.22)	
Lower‐outer	106 (7.23)	105 (7.16)	
Axillary tail	3 (0.20)	5 (0.34)	
Overlapping lesion	349 (23.79)	374 (25.49)	
Molecular subtype			0.7547
HR+/HER2−	1234 (84.12)	1232 (83.98)	
HR+/HER2+	107 (7.29)	104 (7.09)	
HR−/HER2+	25 (1.70)	33 (2.25)	
TNBC	101 (6.88)	98 (6.68)	

Abbreviations: HER2−, HER2‐negative; HER2+, HER2‐positive; HR−, HR‐negative; HR+, HR‐positive; IDC + ILC, invasive duct and lobular carcinoma; IDC, invasive ductal carcinoma; ILC, invasive lobular carcinoma; TNBC, triple‐negative breast cancer.

^a^ The tumor histology of 156 patients (10.63%) at left and 175 patients (11.93%) at right belonged to other types.

^b^ The malignancy grade of 64 patients (4.36%) at left and 43 patients (2.93%) at right were unknown.

^c^ The tumor location of 200 patients (13.63%) at left and 214 patients (14.59%) at right were unknown.

**TABLE 5:** Concordance of pathologic parameters between left and right tumors in BPBC patients in SEER.

Left (*n* = 1467)	Right (*n* = 1467)				*p* (left vs. right)
Tumor histology^a^	R‐IDC	R‐ILC	R‐IDC + ILC		<0.0001
L‐IDC	724 (75.57%)	83 (41.71%)	62 (45.93%)		
L‐ILC	103 (10.75%)	79 (39.70%)	25 (18.52%)		
L‐IDC + ILC	51 (5.32%)	22 (11.06%)	36 (26.67%)		
TNM stage	R‐0/I	R‐II	R‐III		<0.0001
L‐0/I	532 (60.94%)	234 (51.66%)	56 (39.72%)		
L‐II	265 (30.36%)	164 (36.20%)	48 (34.04%)		
L‐III	76 (8.71%)	55 (12.14%)	37 (26.24%)		
HER2 status	R‐HER2+	R‐HER2−			<0.0001
L‐HER2+	38 (27.74%)	94 (7.07%)			
L‐HER2−	99 (72.26%)	1236 (92.93%)			
ER status	R‐ER+	R‐ER‐			<0.0001
L‐ER+	1245 (93.82%)	87 (62.14%)			
L‐ER−	82 (6.18%)	53 (37.86%)			
PR status^b^	R‐PR+	R‐PR‐			<0.0001
L‐PR+	1015 (86.68%)	154 (53.29%)			
L‐PR−	156 (13.32%)	135 (46.71%)			
Molecular subtype	R‐HR+/HER2−	R‐HR+/HER2+	R‐HR‐/HER2+	R‐TNBC	<0.0001
L‐HR+/HER2−	1090 (88.47%)	68 (65.38%)	22 (66.67%)	54 (55.10%)	
L‐HR+/HER2+	76 (6.17%)	26 (25.00%)	3 (9.09%)	2 (2.04%)	
L‐HR−/HER2+	11 (0.89%)	5 (4.81%)	4 (12.12%)	5 (5.10%)	
L‐TNBC	55 (4.46%)	5 (4.81%)	4 (12.12%)	37 (37.76%)	

Abbreviations: ER−, ER‐negative; ER+, ER‐positive; HER2−, HER2‐negative; HER2+, HER2‐positive; HR−, HR‐negative; HR+, HR‐positive; IDC + ILC, invasive duct and lobular carcinoma; IDC, invasive ductal carcinoma; ILC, invasive lobular carcinoma; L, left; PR−, PR‐negative; PR+, PR‐positive; R, right; TNBC, triple‐negative breast cancer.

^a^ 282 patients with other types of tumor histology at left or right were unevaluated.

^b^ Seven patients with unknown PR status at left or right were unevaluated.

To compare BPBC with UBC in SEER, 2874 patients with UBC were randomly matched from 2005 to 2015, and the differences between BPBC and UBC were also investigated. Tumors with lobular components were more frequently observed in BPBC patients than in UBC patients (36.26% vs. 14.54%, *p* < 0.0001, Figure 2B). Compared with BPBC patients, the median age of UBC patients was younger (64 vs. 61 years, *p* < 0.0001, Figure 2C). In addition, there were more ER‐positive tumors in BPBC patients than in UBC patients (90.63% vs. 82.60%, *p* < 0.0001), and HER2‐positive status was more prevalent in UBC patients than in BPBC patients (12.94% vs. 9.17%, *p* < 0.0001).

**FIGURE 3:**
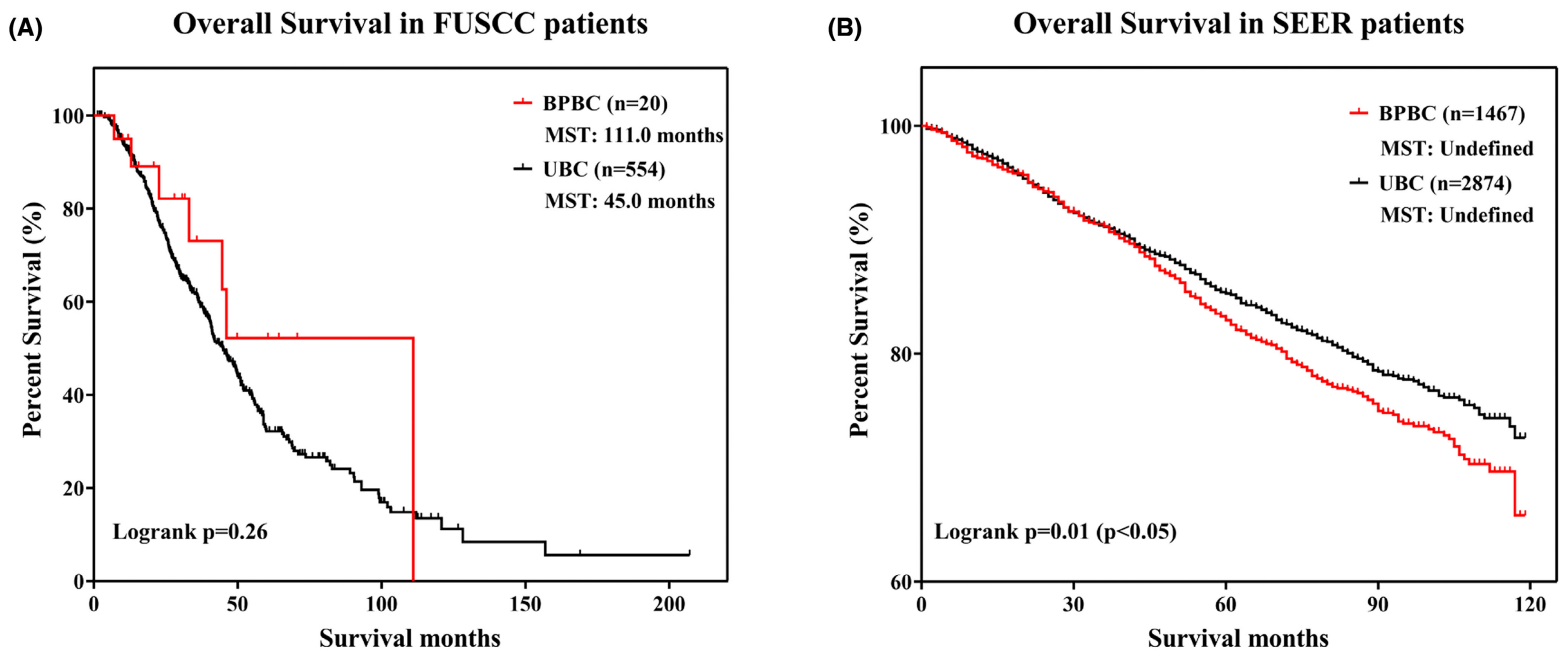
Comparison of prognosis between bilateral primary breast cancer and unilateral breast cancer. (A) Kaplan–Meier curves of OS in Fudan University Shanghai Cancer Center patients. (B) Kaplan–Meier curves of OS in SEER patients. MST, median survival time.

The OS results of patients in SEER showed that the patients with BPBC had a shorter OS than the patients with UBC (hazard ratio: 1.19, 95% CI: 1.03–1.36, *p* < 0.05) (Figure 3).

## 4 DISCUSSION

In this study, we analyzed the clinicopathologic characteristics of patients with BPBC in our NGS database and the SEER database, and explored the mutation profiles of BPBC patients with metastatic disease for the first time.

Our study found that tumors in BPBC patients had more lobular components and ER‐positive status than those in patients with UBC, based on which we hypothesized that endocrine therapy may be important for BPBC patients. Some studies have indicated that the majority of lobular breast cancers are ER‐positive, which might contribute to the greater number of ER‐positive tumors in BPBC patients.^11^, ^12^ It has been suggested that ILC might predict the development of BPBC.^13^ In the SEER database, 312 of 1185 (26.33%) BPBC patients were presented with ILC tumors on either side. However, in our NGS database, 2 of 19 (10.53%) BPBC patients presented with unilateral ILC, and the histology of tumors in most patients was IDC, which may be because only metastatic patients were enrolled in our database and IDC is more prone to metastasis than ILC. Notably, in two patients, the pathology of the metastatic lesions was TNBC, although the bilateral primary lesions were HR+/HER2− tumors, which emphasizes the importance of rebiopsy of metastatic lesions, even when the molecular subtypes of left and right tumors are same in BPBC.

It was indicated that the prognosis of BPBC patients was worse than that of UBC patients in the SEER database, but in our NGS database, we were unable to explore the significance due to our limited number of BPBC patients. Based on the previous studies, the prognosis of BPBC is controversial. Jan J. Jobsen et al.'s study showed that shorter OS in 41 patients with SBPBC than in those with UBC, but no difference in disease‐specific survival was observed in Stefan M. Schmid et al.'s study.^14^, ^15^ A meta‐analysis consisting of 15 studies including 2912 BPBC patients and 72,302 UBC patients also suggested that compared to patients with UBC, patients with SBPBC had worse OS, while MBPBC patients had similar survival to UBC patients.^16^

In addition, it was reported that 16.7%–43% of BPBC patients had a family history of breast cancer in first‐degree relatives.^17^, ^18^ BPBC patients with a family history of breast cancer had a higher risk of germline *BRCA1/2* mutations than those patients without a family history.^19^, ^20^ Another study also demonstrated that germline *BRCA1/2* mutations can increase the risk of developing contralateral breast cancer.^21^ The frequency of germline *BRCA1/2* mutations in patients with BPBC was 10.4%–68.4% in previous studies,^4^, ^22^, ^23^ but our study found only one BPBC patient (5.9%) with a germline *BRCA2* mutation. In a large study of 313 SBPBC patients, in which most of tumors (87.6%) were luminal subtype, 4 of 20 patients were found with germline *BRCA1/2* mutations.^24^ No statistical differences were found between this study and our NGS data due to the small number of patients with sequencing data. In addition to the genes already indicated, there are still other minor susceptibility genes that may play a role in the formation of BPBC, such as *PALB2* and *RAD51C*. A recent study demonstrated that non‐*BRCA* gene germline mutations are found in breast cancer patients with high risk factors, including BPBC. In this study, 5 of 13 (38.5%) BPBC patients had germline *BRCA1/2* mutations, and 1 of 13 (7.7%) BPBC patients carried a non‐BRCA gene mutation (*RAD51C*) related to homologous recombination repair function.^25^ It has also been shown that germline mutations of *PALB2*, an important breast cancer susceptibility gene, conferred an increasing risk of having contralateral breast cancer (hazard ratio, 2.9, *p* = 0.006), and another study indicated that among 235 female patients with pathogenic variants, 28 patients had mutations of *PALB2*, and the bilateral mastectomy rate of these 28 patients was 61%.^26^, ^27^, ^28^ Besides, germline mutations of *CHEK2*, *CDH1*, *TP53*, *NBN*, and *MRE11A* in BPBC patients were also reported, suggesting that patients with these germline mutations should be aware of the development of the BPBC.^4^, ^5^ Although our study had no BPBC patients with microsatellite instability‐high (MSI‐H) status (data not shown), a previous study showed that 10.0% (6/60) of MBPBC patients had MSI‐H status and 1/22 (5.0%) patients with SBPBC was identified to have MSI‐H status.^29^ Immune checkpoint inhibitors could be used for MSI‐H cancers, which is a reminder to carry out comprehensive genetic tests and MSI status tests of BPBC patients to provide the potential to develop more available treatment strategies.^30^ For somatic mutations, our results show that BPBC patients have similar mutation spectra among common mutations compared with UBC patients, which suggests that BPBC patients can be treated as UBC patients in terms of somatic mutations, but more data are needed to confirm this. Moreover, the large study enrolled 313 SBPBC patients suggested that somatic genetic aspects in tumors on the left and right sides of SBPBC patients were independent.^24^

Undeniably, there are some limitations of our study. First, because the tested genes in different panels were different, we could only analyze 62 genes shared by all panels in order to avoid biases. Furthermore, the two databases could not be compared with each other because patients were at the early stage in SEER and at the advanced stage in FUSCC. More importantly, the number of our BPBC patients in FUSCC was small, which prevented us from making a comparison with UBC patients. Our study only provided some perspectives for further intensive studies.

In conclusion, our findings demonstrated that there were strong correlations of clinicopathologic features in BPBC between the left and right tumors, and more lobular components were found in BPBC. Common germline mutations, such as *BRCA1/2* and *CHEK2*, were not as high as expected, which suggested that other mutations might play a crucial role in BPBC. Although the mutation spectrum of somatic genes in BPBC was similar to that in UBC, more studies are needed.

## AUTHOR CONTRIBUTIONS

**Bin Li:** Formal analysis (equal); investigation (lead); visualization (equal); writing – original draft (lead); writing – review and editing (equal). **Weiqi Xu:** Formal analysis (equal); methodology (equal); validation (lead). **Jianing Cao:** Investigation (supporting); validation (equal). **Duancheng Guo:** Investigation (supporting). **Zhonghua Tao:** Data curation (lead); funding acquisition (supporting); project administration (lead); resources (equal). **Juan Jin:** Conceptualization (equal); formal analysis (equal); methodology (equal); resources (equal); software (lead); writing – review and editing (equal). **Xichun Hu:** Conceptualization (equal); funding acquisition (lead); resources (equal); supervision (lead).

## FUNDING INFORMATION

This work was supported by grants from National Natural Science Foundation of China (81903084), Three‐Year Action Plan for Clinical Applications (SHDC2020CR3027B) and National Major Science and Technology Projects of China (2020ZX09201‐013).

## CONFLICT OF INTEREST STATEMENT

The authors have no relevant financial or non‐financial interests to disclose.

## ETHICS STATEMENT

The studies involving human participants were reviewed and approved by Ethics Committee of Fudan University Shanghai Cancer Center (Approval number: 1705172‐9). The participants provided their written informed consent to participate in this study.

## Supporting information


Figure S1.

Figure S2.

Table S1.

Table S2.

## Data Availability

The data can be made available to researchers on request (subject to a review of secrecy). Requests for data can be sent to corresponding authors: Xichun Hu, e‐mail: huxichun2017@163.com, Zhonghua Tao, e‐mail: drtaozhh@126.com, Juan Jin, e‐mail: medjinjuan@126.com.

## References

[1] Siegel RL , Miller KD , Fuchs HE , et al. Cancer statistics, 2022. CA Cancer J Clin. 2022;72(1):7‐33. 35020204 10.3322/caac.21708

[2] Sakai T , Ozkurt E , Desantis S , et al. National trends of synchronous bilateral breast cancer incidence in the United States. Breast Cancer Res Treat. 2019;178(1):161‐167. 31325072 10.1007/s10549-019-05363-0

[3] Li X , Wang Y , Pan B , et al. Clinical characteristics and clinicopathological correlations of bilateral breast cancer in China: a multicenter study from Chinese Society of Breast Surgery (CSBrS‐006). Chin J Cancer Res. 2021;33(1):27‐32. 33707925 10.21147/j.issn.1000-9604.2021.01.03 PMC7941691

[4] Shin H , Lee H , Yoo T , et al. Detection of germline mutations in breast cancer patients with clinical features of hereditary cancer syndrome using a multi‐gene panel test. Cancer Res Treat. 2020;52(3):697‐713. 32019277 10.4143/crt.2019.559 PMC7373875

[5] Nizic‐Kos T , Krajc M , Blatnik A , et al. Bilateral disease common among Slovenian CHEK2‐positive breast cancer patients. Ann Surg Oncol. 2021;28(5):2561‐2570. 33030641 10.1245/s10434-020-09178-y

[6] Hammond MEH , Hayes DF , Wolff AC , et al. American Society of Clinical Oncology/College of American Pathologists Guideline Recommendations for Immunohistochemical testing of estrogen and progesterone receptors in breast cancer. J Oncol Pract. 2010;6(4):195‐197. 21037871 10.1200/JOP.777003 PMC2900870

[7] Wolff AC , Hammond MEH , Hicks DG , et al. Recommendations for human epidermal growth factor receptor 2 testing in breast cancer: American Society of Clinical Oncology/College of American Pathologists Clinical Practice Guideline Update. Arch Pathol Lab Med. 2014;138(2):241‐256. 24099077 10.5858/arpa.2013-0953-SA PMC4086638

[8] Tao Z , Li T , Feng Z , et al. Characterizations of cancer gene mutations in Chinese metastatic breast cancer patients. Front Oncol. 2020;10:1023. 32695676 10.3389/fonc.2020.01023 PMC7338574

[9] Wang M , Chen X , Dai Y , et al. Concordance study of a 520‐gene next‐generation sequencing‐based genomic profiling assay of tissue and plasma samples. Mol Diagn Ther. 2022;26(3):309‐322. 35305253 10.1007/s40291-022-00579-1

[10] Tao Z , Liu J , Li T , et al. Profiling receptor tyrosine kinase fusions in Chinese breast cancers. Front Oncol. 2021;11:741142. 34650924 10.3389/fonc.2021.741142 PMC8506003

[11] Mccart RA , Foong S , Kutasovic JR , et al. The genomic landscape of lobular breast cancer. Cancers (Basel). 2021;13(8):1950. 33919581 10.3390/cancers13081950 PMC8073944

[12] Pramod N , Nigam A , Basree M , et al. Comprehensive review of molecular mechanisms and clinical features of invasive lobular cancer. Oncologist. 2021;26(6):e943‐e953. 33641217 10.1002/onco.13734 PMC8176983

[13] Mejdahl MK , Wohlfahrt J , Holm M , et al. Synchronous bilateral breast cancer: a nationwide study on histopathology and etiology. Breast Cancer Res Treat. 2020;182(1):229‐238. 32441019 10.1007/s10549-020-05689-0

[14] Jobsen JJ , van der Palen J , Ong F , et al. Bilateral breast cancer, synchronous and metachronous; differences and outcome. Breast Cancer Res Treat. 2015;153(2):277‐283. 26268697 10.1007/s10549-015-3538-5

[15] Schmid SM , Pfefferkorn C , Myrick ME , et al. Prognosis of early‐stage synchronous bilateral invasive breast cancer. Eur J Surg Oncol. 2011;37(7):623‐628. 21628090 10.1016/j.ejso.2011.05.006

[16] Pan B , Xu Y , Zhou YD , et al. The prognostic comparison among unilateral, bilateral, synchronous bilateral, and metachronous bilateral breast cancer: a meta‐analysis of studies from recent decade (2008‐2018). Cancer Med. 2019;8(6):2908‐2918. 31038845 10.1002/cam4.2198 PMC6558468

[17] Kheirelseid EA , Jumustafa H , Miller N , et al. Bilateral breast cancer: analysis of incidence, outcome, survival and disease characteristics. Breast Cancer Res Treat. 2011;126(1):131‐140. 20665107 10.1007/s10549-010-1057-y

[18] Baykara M , Ozturk SC , Buyukberber S , et al. Clinicopathological features in bilateral breast cancer. Asian Pac J Cancer Prev. 2012;13(9):4571‐4575. 23167382 10.7314/apjcp.2012.13.9.4571

[19] Narod SA . Bilateral breast cancers. Nat Rev Clin Oncol. 2014;11(3):157‐166. 24492834 10.1038/nrclinonc.2014.3

[20] Pak LM , Gaither R , Rosenberg SM , et al. Tumor phenotype and concordance in synchronous bilateral breast cancer in young women. Breast Cancer Res Treat. 2021;186(3):815‐821. 33242164 10.1007/s10549-020-06027-0

[21] Metcalfe K , Gershman S , Lynch HT , et al. Predictors of contralateral breast cancer in BRCA1 and BRCA2 mutation carriers. Br J Cancer. 2011;104(9):1384‐1392. 21487411 10.1038/bjc.2011.120 PMC3101934

[22] Huang L , Liu Q , Lang G , et al. Concordance of hormone receptor status and BRCA1/2 mutation among women with synchronous bilateral breast cancer. Front Oncol. 2020;10:27. 32117708 10.3389/fonc.2020.00027 PMC7026244

[23] Fasching PA , Yadav S , Hu C , et al. Mutations in BRCA1/2 and other panel genes in patients with metastatic breast cancer‐association with patient and disease characteristics and effect on prognosis. J Clin Oncol. 2021;39(15):1619‐1630. 33780288 10.1200/JCO.20.01200 PMC8274805

[24] Hamy AS , Abecassis J , Driouch K , et al. Evolution of synchronous female bilateral breast cancers and response to treatment. Nat Med. 2023;29(3):646‐655. 36879128 10.1038/s41591-023-02216-8 PMC10033420

[25] Su Y , Yao Q , Xu Y , et al. Characteristics of germline non‐BRCA mutation status of high‐risk breast cancer patients in China and correlation with high‐risk factors and multigene testing suggestions. Front Genet. 2021;12:674094. 34917121 10.3389/fgene.2021.674094 PMC8670232

[26] Vietri MT , Caliendo G , Casamassimi A , et al. A novel PALB2 truncating mutation in an Italian family with male breast cancer. Oncol Rep. 2015;33(3):1243‐1247. 25529982 10.3892/or.2014.3685

[27] Yadav S , Boddicker NJ , Na J , et al. Contralateral breast cancer risk among carriers of germline pathogenic variants in ATM, BRCA1, BRCA, CHEK2, and PALB2. J Clin Oncol. 2023;41(9):1703‐1713. 36623243 10.1200/JCO.22.01239 PMC10022863

[28] Cragun D , Weidner A , Tezak A , et al. Cancer risk management among female BRCA1/2, PALB2, CHEK2, and ATM carriers. Breast Cancer Res Treat. 2020;182(2):421‐428. 32445176 10.1007/s10549-020-05699-y

[29] Kuligina E , Grigoriev MY , Suspitsin EN , et al. Microsatellite instability analysis of bilateral breast tumors suggests treatment‐related origin of some contralateral malignancies. J Cancer Res Clin Oncol. 2007;133(1):57‐64. 16900353 10.1007/s00432-006-0146-0 PMC12160774

[30] Imyanitov EN , Kuligina ES . Systemic investigations into the molecular features of bilateral breast cancer for diagnostic purposes. Expert Rev Mol Diagn. 2020;20(1):41‐47. 31835926 10.1080/14737159.2020.1705157

