# Supplementary figures and images for: A study of clinical and molecular characteristics in bilateral primary breast cancer
Source: Cancer Med. 2023 Jun 9;12(15):15881–92. doi: 10.1002/cam4.6226 (PMC10469734; doi:10.1002/cam4.6226)

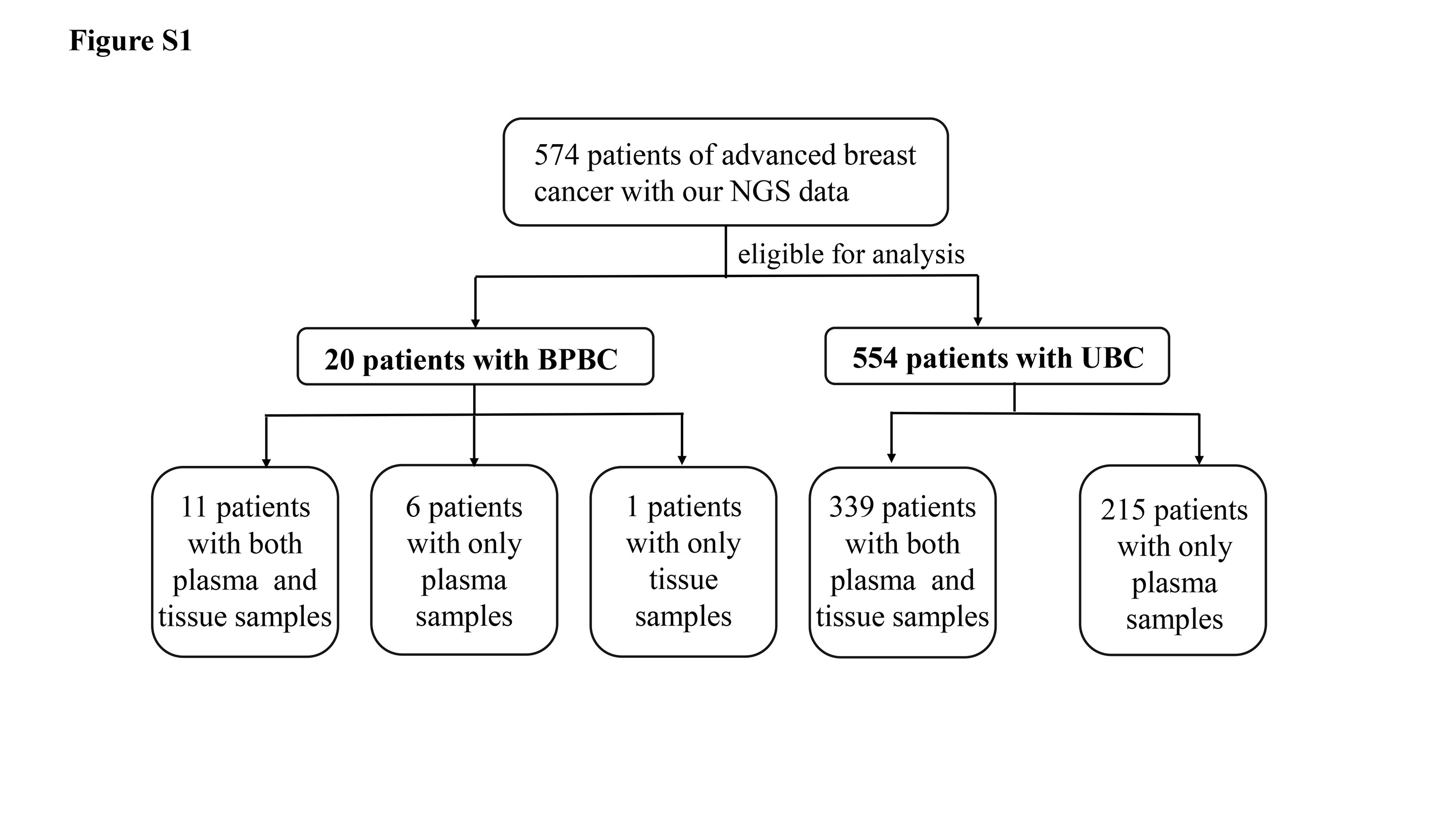

Supplement: Supplementary file 1 — Figure S1. [file CAM4-12-15881-s004.jpg]

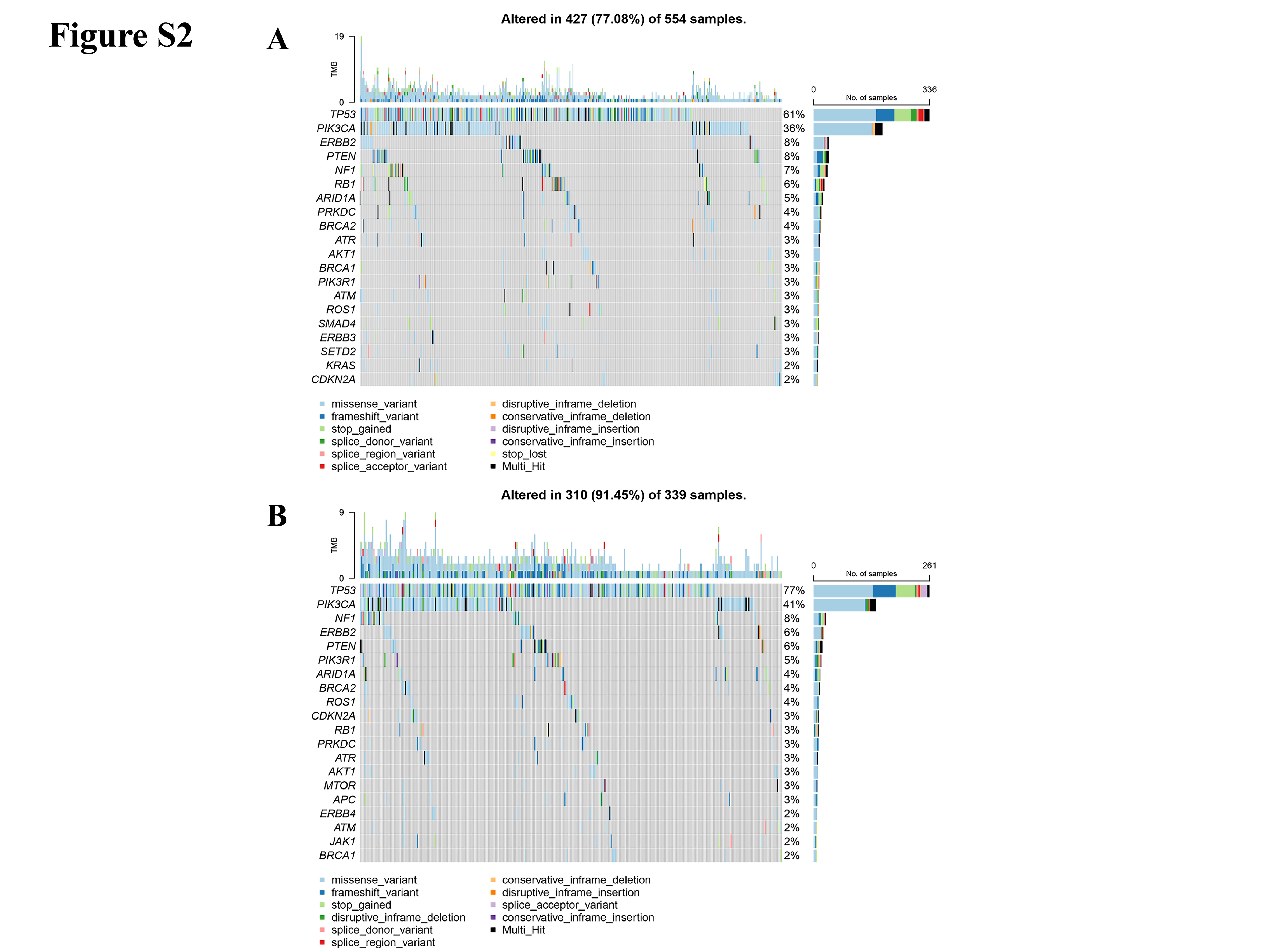

Supplement: Supplementary file 2 — Figure S2. [file CAM4-12-15881-s002.jpg]
