# Supplementary material for: A study of clinical and molecular characteristics in bilateral primary breast cancer
Source: Cancer Med. 2023 Jun 9;12(15):15881–92. doi: 10.1002/cam4.6226 (PMC10469734; doi:10.1002/cam4.6226)
Supplement: Supplementary file 4 — Table S2. [file CAM4-12-15881-s001.docx]

**Supplement Table 2.** **Clinicopathological Characteristics of UBC Patients from FUSCC**

| **Characteristics** | **UBC (n=554)** |
| --- | --- |
| **Age (median, range)** | 45 (21-75) |
| **Family history** |  |
| Yes | 108 (19.50%) |
| No | 333 (60.11%) |
| NA | 113 (20.40%) |
| **Histopathology** |  |
| No lobular component | 518 (93.50%) |
| Lobular component | 16 (2.89%) |
| NA | 20 (3.61%) |
| **The initial metastasis sites** |  |
| Liver | 126 (22.74%) |
| Lung | 214 (38.63%) |
| Bone | 173 (31.23%) |
| **The number of initial metastasis sites** |  |
| <3 | 479 (86.46%) |
| ≥3 | 75 (15.54%) |
| **ER positivity** | 251 (45.31%) |
| **HER2 positivity** | 141 (25.45%) |
| **Previous treatment** |  |
| Adjuvant therapy | 433 (78.16%) |
| Neoadjuvant therapy | 87 (15.70%) |
| NA | 34 (6.14%) |

NA: unknown
